# The GuideLine Implementability Appraisal (GLIA): development of an instrument to identify obstacles to guideline implementation

**DOI:** 10.1186/1472-6947-5-23

**Published:** 2005-07-27

**Authors:** Richard N Shiffman, Jane Dixon, Cynthia Brandt, Abdelwaheb Essaihi, Allen Hsiao, George Michel, Ryan O'Connell

**Affiliations:** 1 Yale Center for Medical Informatics, Yale University School of Medicine, New Haven, CT, USA; 2 Yale School of Nursing, Yale University, New Haven, CT, USA

## Abstract

**Background:**

Clinical practice guidelines are not uniformly successful in influencing clinicians' behaviour toward best practices. Implementability refers to a set of characteristics that predict ease of (and obstacles to) guideline implementation. Our objective is to develop and validate a tool for appraisal of implementability of clinical guidelines.

**Methods:**

Indicators of implementability were identified from the literature and used to create items and dimensions of the GuideLine Implementability Appraisal (GLIA). GLIA consists of 31 items, arranged into 10 dimensions. Questions from 9 of the 10 dimensions are applied individually to each recommendation of the guideline. Decidability and Executability are critical dimensions. Other dimensions are Global, Presentation and Formatting, Measurable Outcomes, Apparent Validity, Flexibility, Effect on Process of Care, Novelty/Innovation, and Computability. We conducted a series of validation activities, including validation of the construct of implementability, expert review of content for clarity, relevance, and comprehensiveness, and assessment of construct validity of the instrument. Finally, GLIA was applied to a draft guideline under development by national professional societies.

**Results:**

Evidence of content validity and preliminary support for construct validity were obtained. The GLIA proved to be useful in identifying barriers to implementation in the draft guideline and the guideline was revised accordingly.

**Conclusion:**

GLIA may be useful to guideline *developers *who can apply the results to remedy defects in their guidelines. Likewise, guideline *implementers *may use GLIA to select implementable recommendations and to devise implementation strategies that address identified barriers. By aiding the design and operationalization of highly implementable guidelines, our goal is that application of GLIA may help to improve health outcomes, but further evaluation will be required to support this potential benefit.

## Background

Tremendous resources have been invested in the development and implementation of clinical practice guidelines over the past 15 years [1-3]. In spite of these efforts, however, guidelines are not uniformly successful in improving care and several instances of implementation failure have been described, some resulting in substantial waste of time and resources [4-6]. In many cases, implementation failures have been related to factors extrinsic to the guideline itself – e.g., organizational and provider-specific obstacles inherent in a particular system of care that interfere with implementation success. In other cases, however, factors intrinsic to the guideline have contributed to implementation failure, e.g., ambiguity, inconsistency, and incompleteness [7,8]. We believe it is particularly important to identify these intrinsic factors, because in many cases they can be ameliorated or fully remedied by guideline authors while the guideline is being developed. If these problems are not captured during guideline development, they must be addressed during implementation.

Guideline *implementation *involves "the concrete activities and interventions undertaken to turn policies into desired results" [9]. We define *implementability *to refer to a set of characteristics that predict the relative ease of implementation of guideline recommendations. Measures of successful implementation include improved adherence to guideline-prescribed processes of care and, ultimately, improved patient outcomes. Indicators of implementability, on the other hand, focus on the ease and accuracy of translation of guideline advice into systems that influence care. In this paper, we describe a tool for appraisal of implementability that is intended to help anticipate barriers to implementation success. We first delineate the process by which indicators of implementability were chosen. Then we describe the steps we took in the preliminary validation of the evolving instrument and show an example of its application. Finally, we discuss how the instrument might be used in practice.

## Methods

### Instrument development

The first step in the measurement of implementability was to define its attributes. From a broad-based literature search, we identified several key papers and book chapters that describe the impact of a variety of factors on success of implementation. The report from the Institute of Medicine [9] included general definitions and several high level constructs relevant to implementation. Thorsen and Mäkelä [10] described critical factors in implementation strategy that facilitate use of guidelines and overcome barriers to adoption. Solberg et al. asked expert implementers about implementation success factors and identified 83 variables grouped into 5 clusters [11]. Applying diffusion of innovation theory, Grilli and Lomas identified guideline complexity, trialability, and observability as critical factors for successful implementation [12]. Grol and colleagues found that vagueness, controversy, demand for a change in routines, and absence of an evidence-base differentiated guidelines that were not followed from those that were [13]. Finally, we examined 3 instruments for appraisal of guideline quality – Cluzeau's 37-item instrument [14], the AGREE instrument [15], and Shaneyfelt's Guideline Quality Appraisal Questionnaire [16]– and extracted factors from each that addressed implementability.

The authors eliminated redundant factors, i.e., those that appeared in several sources or represented concepts that were subsumed by others, through open discussion and consensus. Factors that indicated guideline quality but not implementability were excluded. We decided early on to focus the GLIA on factors that were intrinsic to the guideline, because they could be addressed centrally by a guideline development committee. Thus, we eliminated many factors related to Solberg's medical group characteristics, organizational capability for change, infrastructure for implementation, and external environment. Extrinsic items relating to a recommendation's effect on the process of care and items relating to the novelty or innovation of a guideline statement were retained in the instrument because developers can anticipate these barriers and offer potential strategies for implementation success.

All remaining factors were grouped into categories of related constructs, hereafter referred to as dimensions. We then devised specific questions to characterize each dimension and phrased them so that negative responses identified barriers. These questions ultimately became items of the instrument. We iteratively refined the items, further clarified definitions, and re-categorized items into the most appropriate dimensions.

### Validation

To explore the construct of implementability and its measurement, we carried out a series of validation activities. The steps are summarized in Table 2 and are described below in the sequence in which they were carried out. Concomitant with these validation activities, the GLIA instrument underwent iterative refinement and revision.

**Table 2:** Summary of validation activities.

**Step**	**Process**	**Purpose or Validity Type**	**Results**
1	Ranking of implementability of 3 recommendations by experts at COGS	Validity of the construct of implementability	Consistent ranking
2	Guideline review with early GLIA versions	To refine GLIA	Demonstrate feasibility of measurement of implementability
3	Expert review of GLIA items and dimensions by HL7 experts	Content validity of GLIA	GLIA items rated generally as relevant and clear. Two new items were added, definitions were clarified, and explanatory material was added.
4	Review of the recommendations ranked in Step 1	Construct validity of GLIA	GLIA assessment of these three guidelines was consistent with experts' rankings of implementability (Step 1). Barriers to implementation were explicitly identified.

## Results

### Final instrument

The table shown in [Additional file 1] summarizes the 10 dimensions of the instrument and their definitions. (The GLIA instrument is available for download at .) Of the 31 items in GLIA, the first dimension (Global) contains 7 items that relate to the guideline as a whole. The remainder of the dimensions focus on the individual recommendation as the unit of implementability, since a single guideline may contain recommendations that vary widely in their implementability. For each GLIA item, each recommendation is rated using one of four response options (see Table 1). Additional comments that explain each response are encouraged. GLIA users should discuss all divergent ratings and try to achieve consensus. Any items scored with "?" should be resolved: often, this requires the help of an expert in the guideline's topic area.

**Table 1:** GLIA response options.

Y	The recommendation meets this criterion fully.
N	The recommendation does not meet this criterion.
?	Rater is unable to address this question because of insufficient knowledge or experience in this area.
NA	Criterion is not applicable to this recommendation.

**Figure 1:**
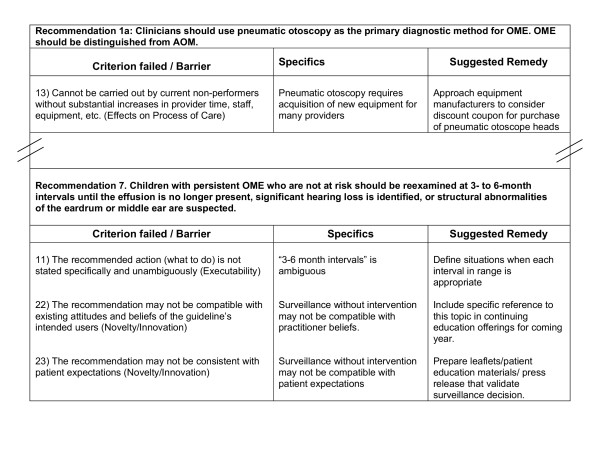
Example of a GLIA Summary Report on draft recommendations from a guideline for diagnosis and management of otitis media with effusion.

When any GLIA item is assigned an "N" response, its corresponding barrier to implementation is recorded on the summary sheet with a brief description of why the recommendation failed the criterion (see Figure 1). Examination of the barriers recorded on the summary sheet should provide an understanding of predicted impediments to implementation of the recommendation. The summary sheet also contains a column where suggested remedies can be described.

### Validation

#### Step 1: Evaluation of implementability as a concept that is understood by experts

In April 2002, the Conference on Guideline Standardization (COGS) brought together 23 national and international leaders in guideline development, dissemination and implementation [17]. This gathering provided the opportunity to explore the concept of implementability. We selected 3 recommendations for review by the experts based on the following criteria:

• Each recommendation concerned a common clinical problem generally understood by the attendees.

• The three recommendations as a group represented a broad range of implementation challenges.

Using our own global subjective judgment of implementability, one of the recommendations was considered to be straightforward to implement, another was considered to be quite difficult, and the remaining recommendation was considered to occupy an intermediate position. The recommendations we selected concerned: aspirin therapy in management of acute myocardial infarction [18], diagnosis of urinary tract infection in young children with unexplained fever [19], and evaluation of unexplained syncope [20].

Divided into 2 groups (10 individuals with considerable experience in guideline implementation in one group, 11 individuals experienced in guideline development and dissemination in the other), participants were asked to rank the relative implementability of the guideline recommendations. After discussion, each group came to a consensus ranking of the three recommendations' implementability. These rankings were consistent between the two groups and also with the authors' expectation. Both groups agreed that the aspirin recommendation would be easiest to implement and the syncope algorithm would be most challenging.

This step provided support for our basic assumption that the construct of implementability is real. Moreover, implementability varies among recommendations in a way that can be systematically recognized by experts.

#### Step 2- Initial guideline review with early GLIA versions

The authors next tested an early version of GLIA with a convenience sample of 20 guideline recommendations. These reviews provided our first experience using the GLIA in practice and led us to identify areas of rating controversy. Early attempts to apply a simple numeric scoring system failed because all the items were not of equal importance. In addition, we recognized the advantage of qualitative rating in understanding specific barriers to implementation. We appreciated the importance of including a variety of expertise among members of the GLIA team. This experience led to refinement of the instrument, as well as to a deeper understanding of the optimal process for conducting a review using GLIA.

The 20 replications of our process seemed quite adequate to provide us with assurance that creating an instrument to assess implementability was a feasible goal. They also gave us a view of the problems still to be addressed.

#### Step 3 - content validation: Expert review of GLIA items and dimensions

To investigate the content validity of the draft GLIA, we systematically examined each item's clarity, its relevance to its superordinate dimension, and the comprehensiveness of the GLIA as a whole [21]. We prepared a Questionnaire for Expert Review on which relevance and clarity ratings were scored on a four point scale, with higher numbers representing greater relevance and greater clarity. We distributed the Questionnaire to volunteers attending a Workgroup Meeting of the HL7 Special Interest Group (SIG) on Clinical Guidelines. The SIG includes representatives from academia, vendors of electronic health record software, and healthcare providers, who meet three times each year to discuss standardization of guideline components.

Judgments about the GLIA were obtained from 7 guideline implementers. Mean *relevancy *ratings by item ranged from 2.7 to 4.0. On average, 26 of 30 items were rated as "moderately" or "highly relevant." No reviewer used a relevancy rating of 1 ("not at all relevant") for any item.

Mean *clarity *ratings by item ranged from 2.3 to 4.0. On average, 29 of 30 items were rated as "clear" or "very clear." The correlation of relevancy ratings and clarity ratings by item was .23 (non-significant), indicating that the two ratings of each item provided different information.

Raters were also asked for suggestions about the items and numerous comments were provided. Based on this feedback from implementers, GLIA was again revised. Items that received low clarity or relevance scores were carefully reviewed, definitions were clarified, and explanatory material was added to better communicate the meaning of the item. Based on suggestions from the reviewers, two new items that explored recommendation sequencing and the internal consistency of the guideline were added to the Global dimension.

This process of obtaining judgment data from implementers proved to be a rich source of ideas for refinement of items. It also provided supportive evidence for the general content validity of the developing instrument.

#### Step 4 -Review of ranked recommendations

Next, we used GLIA to formally assess the three recommendations whose implementability had been ranked previously by experts at the COGS Meeting (described above in Step 1). Results using GLIA were consistent with the earlier expert rankings, i.e., more critical implementation barriers were identified in the recommendation ranked least implementable and no barriers were identified in the recommendation ranked most implementable. Moreover, appraisal with GLIA allowed us to itemize specific obstacles, thereby clarifying particular impediments to implementation in contrast to the global subjective evaluation performed in Step 1.

This finding of agreement with expert rankings indicated that GLIA results reflect the construct of implementability as conceptualized by experts. This consistency provided preliminary evidence that supports the construct validity of GLIA assessments.

#### Step 5 – Application to a draft guideline from the American Academy of Pediatrics

To assess the value of application of GLIA in a real world guideline development effort, we applied a late version of the appraisal to a draft clinical guideline for the management of otitis media with effusion (OME) that was in preparation by a joint committee of national professional societies – the American Academy of Pediatrics, the American Academy of Family Physicians, and the American Academy of Otolaryngology. We received an intermediate draft of the guideline for quality appraisal and evaluation of implementability.

After independently rating the draft guideline, we met as a group to discuss the barriers to implementation that we identified. While not every barrier was identified by every rater, no single barrier was uniquely identified. Remarkably little discussion was needed to reach a consensus on anticipated barriers to implementation.

Our report to the guideline authors identified several instances of problems with decidability and executability of individual recommendations. For example, the draft recommended, "During the *initial assessment *of the child with OME, the clinician should document (a detailed set of physical findings)" (italics added). The GLIA report drew attention to the fact that the guideline's users might not consistently determine when in the course of continuous care an assessment is *initial*. Adding to the confusion, the draft guideline text later states that these findings should be ascertained at *every *medical encounter. The recommendation that was ultimately published stated: "Clinicians should document (the physical findings) at each assessment of the child with OME." Vagueness was also inherent in use of the terms "academic risk", "when necessary", and "individualized management" without clear definitions. Following the GLIA report, each of these was clarified in the final guideline publication [22].

GLIA appraisal also identified extrinsic barriers to implementation that were reported to the Joint OME Implementation Committee of the professional societies. These barriers included:

• Recommendations to perform pneumatic otoscopy, tympanometry, hearing screening, and language assessment would require acquisition of new equipment and skills on the part of many physicians.

• Recommendations against prescription of antihistamines, decongestants, antimicrobials and corticosteroids for effusions may not be compatible with patient expectations.

The committee considered these potential barriers in their design of a guideline implementation strategy.

## Discussion

Many clinical guidelines – developed at substantial cost and effort – have proven to be difficult or impossible to operationalize. We developed the GuideLine Implementability Appraisal to facilitate guideline implementation. The instrument is designed to systematically highlight barriers to implementation.

The GLIA is intended to provide feedback about a guideline's *implementability *to two distinct audiences: the authors of the guideline and those individuals who choose guidelines for application within a health care delivery system. As a guideline is being developed, GLIA can provide feedback to guideline authors about potentially remediable defects. Developers may choose to make modifications to the guideline document before it is finalized and disseminated. Implementers can use GLIA to help select a guideline, to identify potential obstacles, and to target efforts toward addressing identified barriers. Thus, GLIA can be used to help select guidelines that are more easily implementable and also to devise implementation strategies that address identified barriers.

Two GLIA dimensions are of particular importance because failure to address them adequately will result in inconsistent implementation [23,24]. Any recommendation that does not clearly communicate what to do (i.e., it fails executability criteria) or when to do it (i.e., fails decidability criteria) is not fully ready for implementation. If possible, guideline authors should revise such a recommendation before it is disseminated for implementation. If problems in decidability and executability are not corrected prior to dissemination, different implementers may well interpret the guideline authors' intent in a discordant manner.

GLIA incorporates an optional dimension – computability – to indicate the ease with which a recommendation might be operationalized in an electronic information system. Guideline implementation strategies – e.g., education, academic detailing, audit and feedback, administrative sanctions – need not necessarily involve computers [25]. Because of the success of electronic implementations in influencing clinician behavior [26-28], the wide variation in electronic information systems, and the current lack of guidance regarding computability, these extrinsic considerations were retained in the instrument. Items in the *computability *dimension address the availability of data to trigger the recommendation, the level of specificity of the triggers and recommended actions, and whether there is a clear path from recommendation to electronic implementation.

Implementability must be differentiated from guideline quality. Quality assessments relate primarily to determining the scientific validity of guidelines and, generally, quality is assessed for the guideline as a whole. Implementability, on the other hand, is one component of guideline quality, but its assessment is applied largely to individual recommendations within a guideline.

In a comprehensive review of 13 tools for guideline quality appraisal, Graham [29] identified instruments developed by Cluzeau [14] and Shaneyfelt et al [16] as the best developed. Since that time, the AGREE Instrument [15] has emerged as the leading exemplar of guideline quality appraisal and it has been endorsed by the Guidelines International Network [30]. Application of GLIA can complement quality appraisal in identifying guideline deficiencies. Several GLIA items overlap with items in the AGREE scale. However, to the best of our knowledge, GLIA is the only tool that emphasizes implementation concerns at the level of the individual recommendation.

Application of GLIA to measurement of decidability and executability requires that users translate guideline recommendations into statements comprising conditions and actions [31]. Training and practice may be required to assure consistency of logical analysis.

Application of GLIA requires team effort and a consequent resource investment. The team that applies GLIA should include members with skills in guideline implementation as well as members with specific understanding of the clinical domain. Our experience has demonstrated that rating a guideline that contains 15 recommendations might require several hours of an individual's time. Additional time must be spent in resolving divergent ratings, although this effort usually yields an improved understanding of implementation issues. We are currently developing an electronic version of GLIA that will provide a more efficient means of rating, scoring, and reporting results.

During development, GLIA is best applied once evidence has been synthesized and draft recommendations have been formulated. When applied too late in the authoring process, GLIA may have limited impact because authors may have already become committed to recommendations as written and thus not open to making modifications. Assuring that guideline authors understand the importance of implementability early on may help to overcome premature commitment.

The classic challenge of instrument development is to arrive at the correct number of items to minimize burden and avoid redundancy, while including a sufficient number to be comprehensive. GLIA contains 7 global items that are applied once to each guideline, 20 items that are applied to each rated recommendation, and 4 optional items rating computability that are applicable when an electronic implementation is planned. Further use of GLIA is likely to result in clarification and perhaps modification of the number of items.

### Limitations

We have performed a series of activities to provide preliminary evidence of GLIA's validity. However, neither the inter-rater reliability of GLIA, the test-retest reliability, the factor structure of the dimensions, nor its predictive validity has yet been established. Plans for this testing are underway.

It should be noted that the authors performed the activities described as Step 4. Until verified by an independent panel, these results should be considered preliminary and subject to potential bias.

In addition, GLIA addresses primarily factors intrinsic to the guideline. It is clear that extrinsic factors are critical in a successful implementation. Future extensions to GLIA will help to identify extrinsic barriers to implementation.

## Conclusion

The GuideLine Implementability Appraisal provides a tool designed to help developers and implementers better understand and anticipate barriers to successful implementation. By aiding the design and operationalization of highly implementable guidelines, our goal is that application of GLIA may help improve health outcomes. Demonstration of GLIA's effectiveness will require prospective testing.

## Competing interests

The author(s) declare that they have no competing interests.

## Authors' contributions

RS conceived the instrument, performed the literature review, participated in all phases of instrument development and validation, and edited the manuscript.

JD participated in all phases of instrument development and validation and edited the manuscript.

CB, AE, AH, GM, and RO participated in all phases of instrument development and validation and reviewed the manuscript.

## Pre-publication history

The pre-publication history for this paper can be accessed here:



## Supplementary Material

Additional File 1 GLIA dimensions and example items Click here for file
