# Supplementary material for: The GuideLine Implementability Appraisal (GLIA): development of an instrument to identify obstacles to guideline implementation
Source: BMC Med Inform Decis Mak. 2005 Jul 27;5:23. doi: 10.1186/1472-6947-5-23 (PMC1190181; doi:10.1186/1472-6947-5-23)
Supplement: Additional File 1 — GLIA dimensions and example items [file 1472-6947-5-23-S1.doc]

## GLIA dimensions and their characteristics, including example items.

| **Dimension** | Definition | **# of Items** | **Example Item** |
| --- | --- | --- | --- |
| Global | General characteristics of the guideline as a whole | 7 | 1) Do the organization(s) and author(s) who developed the guideline have credibility with the intended users of the guideline? |
| Decidability | Precisely under what conditions to do something | 3 | 8) Would the guideline's intended audience consistently determine whether each condition in the recommendation has been satisfied? That is , is each and every condition described clearly enough so that reasonable practitioners would agree when the recommendation should be applied? |
| Executability | Exactly what to do under the circumstances defined | 2 | 12) Is sufficient detail provided or referenced (about how to do it) to allow the intended audience to perform the recommended action, given their likely baseline knowledge and skills? |
| Presentation and formatting | Degree to which the recommendation is easily recognizable and succinct | 2 | 15) Is the recommendation easily identifiable, e.g., summarized in a box, typed in bold, underlined, presented as an algorithm, etc.? |
| Measurable outcomes | Degree to which the guideline identifies markers or endpoints to track the effects of implementation of this recommendation | 2 | 18) Can criteria be extracted from the guideline that will permit outcomes of this recommendation to be measured? |
| Apparent validity | Degree to which the recommendation reflects the intent of the developer and the strength of the evidence | 2 | Is the quality of evidence that supports the recommendation explicitly stated? |
| Flexibility | Degree to which a recommendation permits interpretation and allows for alternatives in its execution | 4 | 24) Does the recommendation specify patient or practice characteristics (clinical and non-clinical) that require (or permit) individualization? For example, immediate angioplasty and MR imaging may not be available in all settings. |
| Effect on process of care* | Degree to which the recommendation impacts upon the usual workflow of a care setting | 2 | 13) Can the recommendation be carried out by current non-performers without substantial increases in provider time, staff, equipment, etc.? |
| Novelty/innovation* | Degree to which the recommendation proposes behaviors considered unconventional by clinicians or patients | 3 | 21) Can the recommendation be performed by the guideline’s intended users without acquisition of new competence (knowledge, skills)? |
| Computability*÷ | Ease with which a recommendation can be operationalized in an electronic information system | 4 | 28) Are all patient data needed for this recommendation available electronically in the system in which it is to be implemented? |

*Dimension requires consideration of extrinsic factors
